# The Envelope

**Published:** 1871-01

**Authors:** 


### THE ENVELOPE.

Our friends will find the yearly envelope, with blank, for continuing their subscription to the BISTOURY. All that is necessary to be done is to enclose fifty cents in the blank; after having filled it out properly, place it in the Post Office with a three cent stamp affixed, and your subscription is paid up in full, for another year. We will give you credit for the same on the inside of the cover of the next issue.

To those who have paid nothing to the BISTOURY, and who have been receiving it for the past two years,—there are quite a number of you—we want you to enclose *one dollar*, and ask us to discontinue the journal. We will be happy to do so, as we have no difficulty in finding more appreciative subscribers.

We hope our friends will pay up promptly, and enable us to make several contemplated improvements in the appearance of our journal.

